# Maternal AA/EPA Ratio and Triglycerides as Potential Biomarkers of Patients at Major Risk for Pharmacological Therapy in Gestational Diabetes

**DOI:** 10.3390/nu14122502

**Published:** 2022-06-16

**Authors:** Chiara Maria Soldavini, Gabriele Piuri, Gabriele Rossi, Paola Antonia Corsetto, Linda Benzoni, Valeria Maggi, Giulia Privitera, Angela Spadafranca, Angela Maria Rizzo, Enrico Ferrazzi

**Affiliations:** 1 Obstetrics Unit, Department of Woman Child and Newborn, Fondazione IRCCS Ca’ Granda, Ospedale Maggiore Policlinico, 20122 Milan, Italy; gabriele.piuri@me.com (G.P.); gabriele.rossi@policlinico.mi.it (G.R.); valeria.maggi@policlinico.mi.it (V.M.); giulia.privitera@policlinico.mi.it (G.P.); angela.spadafranca@gmail.com (A.S.); enrico.ferrazzi@unimi.it (E.F.); 2 Department of Pharmacological and Biomolecular Sciences, University of Milan, 20134 Milan, Italy; paola.corsetto@unimi.it (P.A.C.); linda.benzoni@unimi.it (L.B.); 3 Department of Clinical and Community Sciences, University of Milan, 20122 Milan, Italy

**Keywords:** AA/EPA ratio, inflammation, gestational diabetes, nutraceuticals, oxidative-stress, platelet-activating factor

## Abstract

Gestational diabetes mellitus (GD) is characterized by glycemic and lipid metabolism alterations in an environment of low-grade inflammation. Our trial aimed to assess the effect of nutraceutical supplements (omega-3 fatty acids, anthocyanins, and alpha-cyclodextrins) in GD patients and evaluate the role of anthropometric, metabolic, and inflammatory parameters as biomarkers to identify subjects who require pharmacological hypoglycemic treatment during gestation. Pregnant women with GD at 24–28 weeks of gestation were enrolled in a double-blind trial and randomized to receive either nutraceutical supplements or a placebo for 12 weeks. No statistically significant differences were observed between the two groups in blood and urine measurements of metabolic, inflammatory, and antioxidant parameters. In the whole cohort, pre-pregnancy BMI and anthropometric measurements were significantly different in patients who required pharmacological intervention. These patients showed higher triglycerides, CRP, and insulin levels and gave birth to newborns with significantly higher weights. Subjects with a greater AA/EPA ratio had higher PAF levels and gave birth four days earlier. In conclusion, one-to-one nutritional coaching and poor compliance with nutraceutical supplementation might have outweighed the impact of this intervention. However, triglyceride concentration and the AA/EPA ratio seems to be a biomarker for higher inflammatory levels and GD candidates for pharmacological treatment. An adequate assumption of omega-3 in women with GD, either by a controlled diet or by nutraceutical supplementation, reduces the need for pharmacological therapy.

## 1. Introduction

Low-grade chronic inflammation plays a pivotal role in the pathogenesis and maintenance of pathological conditions such as obesity, metabolic syndrome, diabetes, and other chronic diseases [1]. The dietary treatment has been shown to effectively reduce the production of pro-inflammatory eicosanoids, thus representing a remarkable non-pharmacological approach to these conditions [2]. Recently, growing interest in the benefits of polyphenols of plant origin and omega-3 fatty acids has led scientific research to evaluate their antioxidant and anti-inflammatory effects in in vitro and in vivo studies [3,4,5,6].

Since pregnancy itself represents a pro-inflammatory state, dietary and nutraceutical approaches to reduce reactive oxygen species (ROS) and eicosanoid production have been proposed to avoid more intrusive pharmacological treatments and improve pregnancy outcomes for the mother and the child [7].

Gestational diabetes mellitus (GD) represents a common pregnancy complication linked to high levels of inflammation [8]. Changes driven by the fetoplacental unit in the maternal organism led to metabolic stress over the insulin, glucose, and lipid homeostatic systems [9,10]. Patients with GD show a damaged, altered metabolism; GD has been described as a clinical phenotype of an accelerated metabolic syndrome characterized by anti-insulin effects and dyslipidemia. This condition has been shown to negatively impact the health of both the mother and the offspring [11]. In fact, in the long term, the mother displays an increased risk of developing obesity, type 2 diabetes, hypertension, and heart disease [12]. Moreover, the critical intrauterine environment promotes predisposition to childhood obesity and diabetes through epigenetic fetal programming [13,14,15].

Multiple evidence suggests that polyunsaturated fatty acids (PUFA) may influence physiological and pathological status modulating metabolism, inflammation, and oxidative process. In several studies, metabolic differences in maternal and fetal PUFA have been reported between mothers with and without GD [16]. Both omega-3 and omega-6 fatty acids are involved in the process of inflammation and its resolution, generating eicosanoids and bioactive lipid mediators. The arachidonic acid/eicosapentaenoic acid ratio (AA/EPA) is a relevant biomarker of PUFA status, indicating the balance between omega-6 and omega-3 and their derived bioactive lipid mediators [17,18,19]. This ratio represents a sensitive marker of dietary habits, and it is useful to follow omega-3 integration compliance during clinical studies. FA composition of the blood is a biomarker of dietary fat intake, with plasma phospholipids reflecting short-term dietary fat intake compared to red blood cell membrane phospholipids that are modified after one or two months of fat intake [20,21].

The development of GD could be induced by an increase in the inflammatory condition connected to inflammatory cytokines and oxidative stress. Platelet-activating factor (PAF) is an inflammatory mediator and could contribute to GD pathogenesis and pre-eclampsia pathogenesis. PAF levels increase in women with GD from diagnosis to 12 weeks after the diagnosis and are positively correlated with glycated hemoglobin levels and the HOMA index. An increase in PAF values describes a proinflammatory condition of GD and could worsen a condition of oxidative stress and metabolic impairment [22].

The first aim of the present randomized double-blind trial was to evaluate the metabolic effects on GD patients of anti-inflammatory, antioxidant nutraceutical supplementation with omega-3 fatty acids, anthocyanins, and alpha-cyclodextrins associated with appropriate nutritional coaching versus nutritional coaching and placebo.

The secondary aim was to assess in the whole cohort the role of metabolic and inflammatory markers to predict the severe cases of GD who require drug therapy in addition to an appropriate diet.

## 2. Materials and Methods

### 2.1. Study Design and Population

From May 2018 to March 2020, we conducted a randomized, double-blind, placebo-controlled monocentric trial at Mangiagalli High Risk Maternity Centre, Fondazione IRCCS Cà Granda Ospedale Maggiore Policlinico, Milan, Italy. The study was approved by the Ethical Committee Milan Area 2 (PRE.D.I.P.2, project identification code 4004, approval number 126 on 28 March 2018).

We included patients between 24 and 28 weeks of gestation who presented a positive result to an Oral Glucose Tolerance Test with 75 gr glucose performed after 24 gestational weeks, according to the International Association of Diabetes and Pregnancy Study Group (IADPSG) recommendations (at least one of the following criteria: baseline glycemia ≥ 92 mg/dL, 1-h glycemia ≥ 180 mg/dL, 2-h glycemia ≥ 153 mg/dL) [23]. Moreover, maternal age should be ≥ 18 years. Eligible women were recruited during their first obstetric visit at the maternal-fetal medicine outpatient clinics reserved for GD patients. Written formal consent was obtained from all the patients enrolled.

We excluded multiple pregnancies, fetal malformation, maternal diseases (type 1 and type 2 diabetes, hypothyroidism and hyperthyroidism, immunological disorders), and abnormal blood glucose values before 24 weeks of gestation.

### 2.2. Randomization and Treatment Allocation

At recruitment, women were randomly assigned to receive either anti-inflammatory nutraceutical supplements (intervention group) or placebo (control group) in sequentially labelled, opaque sealed envelopes. A computer-generated random list arranged labels with a target 1:1 allocation ratio. Participants and care providers were blinded to group assignments. Products should be taken daily for at least 70 days until the last study visit performed at 36–39 weeks of gestation.

Anti-inflammatory nutraceutical supplements included the following:

- Omega-3 fatty acids (pills, EnerZona Omega3Rx^®^, Enervit, Italia), at a daily dosage of 2.4 gr at breakfast;
- Anthocyanins (pills, EnerZona Maqui Response Capsule^®^, Enervit, Italia) at a total daily dosage of 108 mg divided into three equal intakes at breakfast, lunch, and dinner;
- Alpha-cyclodextrins (sachets, EnerZona Maqui Response Buste^®^, Enervit, Italia) at a total daily dosage of 15 gr divided into three equal intakes at breakfast, lunch, and dinner.

The supplementation did not present toxicity and its administration is approved under Italian regulations.

The planned sample size of the trial included 30 subjects in the intervention group and 30 subjects in the control group; it was calculated according to the hypothesis of the reduction of median blood glucose values from 87 to 78 mg/dL in patients receiving supplementation, with power 0.97 and alpha error 0.05. The power was selected according to the estimated effect of the nutraceutical supplementation.

### 2.3. Dietary Intervention

All women received nutritional education and a personalized diet by a nutrition expert educator according to the Mediterranean standard diet referrals, the Healthy Eating Plate recommendations of Harvard University School of Public Health, and the reference intake levels of nutrients and energy for the Italian population (LARN) for pregnant women [24,25] The daily total energy intake was distributed in three main meals (breakfast, lunch, and dinner) and in two snacks; caloric intake was calculated according to maternal pre-pregnancy BMI and weight gain. The macronutrient composition was balanced as follows: 45% of total energy from carbohydrates, with simple sugar less than 12%; 25–35% of total energy from fat (less than 7% from saturated fat and 10% from PUFA); protein intake satisfied the pregnant requirement as indicated in Recommended Assumption Levels of Energy and Nutrients for Italian Population with 50% of proteins derived from vegetal source and 50% from animal source. The quality of protein intake was regulated by the following frequencies of consumption: meat, preferable white, 2 times/week; fish, 2–3 times/week, with preference for blue fish for optimal intake of omega-3 fatty acids; legumes, 3–4 times/week; eggs, 2 times/week; cheese, 1–2 times/week; ham, 1 time/week; nuts, 20–30 gr every day. Food with high glycemic index was not allowed. Two servings of fruit and three servings of vegetables were daily advised. Olive oil was indicated as the main culinary lipid. Dietary cholesterol was lower than 200 mg/die and fiber intake was about 30 g/die.

Every woman was invited to meet the dietitian every two weeks to evaluate the weight, the fat mass, and the adherence to diet. Moreover, exercise was strongly recommended, and 30-min daily walk was suggested to accomplish this assignment.

### 2.4. Antenatal Monitoring and Treatment

All participants were instructed to self-monitor capillary blood glucose levels (fasting and 2-h postprandial measurements) at least three times daily using a standard reflectance meter; recordings of these values were submitted to care providers during the obstetric visit.

Patients received twice-weekly antenatal testing, including ultrasonography for fetal growth and well-being maternal weight body fat mass distribution evaluation using skinfold caliper. In addition, dietary counseling was offered at each visit. The last study assessment was performed after at least 70 days of treatment at 36–39 weeks of gestation. Afterward, women underwent the protocol in use at our Institute for monitoring maternal and fetal well-being and delivery.

Personalized regimens of insulin were prescribed if the median blood glucose values were elevated in relation to fetal growth as assessed by ultrasound (fasting glucose level ≥95 mg/dL or 2-h postprandial glucose level ≥120 mg/dL if the fetal abdominal circumference was below the 75° percentile for gestational age; fasting glucose level ≥90 mg/dL or 2-h postprandial glucose level ≥110 mg/dL if the fetal abdominal circumference was equal to or above the 75° percentile for gestational age). Furthermore, obstetric examinations were performed more frequently in case of pharmacological treatment according to the protocol in use at our Institute.

Baseline characteristics, pregnancy, and neonatal outcomes were recorded for all randomized women. For measurements of metabolic, inflammatory, and antioxidant parameters, blood samples were drawn from all patients at recruitment and at the last study visit; in particular, we analyzed plasmatic glycemia, HbA1c, cholesterol, triglycerides, C-reactive protein, cortisol, erythrocyte fatty acid composition, and PAF.

### 2.5. Erythrocyte Fatty Acid Composition

Erythrocytes (RBC) were separated by centrifugation (2000 rpm for 10 min) from heparinized blood drawn at recruitment and at the last study visit performed at 36–39 weeks of gestation and stored at −80 °C until analysis. Cell membranes of RBC (ghosts) were prepared by lysis with hypotonic buffer (phosphate 5 mM, pH 8, EDTA 0.5 mM), precipitated by centrifugation, and washed several times to eliminate hemoglobin residues.

Ghost lipids were extracted with chloroform/methanol according to Folch [26] and the fatty acid composition was determined by direct derivatization with sodium methoxide in methanol and GC-FID analysis as previously described [27]. Each sample was spiked with TG C17:0 as internal standard; a standard mixture containing all fatty acid methylesters (Sigma-Aldrich, St. Louis, MO, USA) was injected for calibration each day.

### 2.6. Plasmatic PAF

The plasmatic PAF was measured via commercial ELISA kits (Human Platelet Activating Factor ELISA Kit, lower range of detection 0.313 ng/mL, sensitivity 0.188 ng/mL, Catalog Number E-EL-H2199, Elabscience, Houston, TX, USA) using the Biomek 4000 ELISA microplate liquid reagent dispensing automation tool (Beckman Coulter, Brea, CA, USA) and the EL405LS ELISA microplate automated washing system (BioTek Instruments, Winooski, VT, USA). The absorbance of each well was read at a wavelength of 450 nm with a Multiskan FC plate reader (Thermo Scientific, Waltham, MA, USA). The average zero standard optical density was subtracted from all absorbances, and a standard curve was generated using a four-parameter logistic (4-PL) curve fit. The concentration in the test sample was calculated through interpolation along the standard curve by multiplying the result by the dilution factor.

### 2.7. Statistical Analysis

Statistical analysis of the data was performed using GraphPad Prism 9 for macOS (GraphPad Software, San Diego, CA, USA. Version 9.3.1 (350)). The median and interquartile range (IQR) were calculated for each variable. The medians were compared using the Mann–Whitney test. The Chi-square test of association was used to evaluate the relationships between categorical variables. A *p*-value < 0.05 was used as the limit of statistical significance.

## 3. Results

Of the 56 eligible subjects, 51 women agreed to participate in this trial. The most common reason for non-participation was a reluctance to undergo treatment. The 51 participants were randomly assigned to either nutraceutical supplements (intervention group, *n* = 26) or placebo (control group, *n* = 25).

Nine patients withdrew from the study (8 in the intervention group and 1 in the placebo group) because of personal issues or gastrointestinal side effects; all of them withdrew within 15 days after randomization. Two participants (1 in the intervention group and 1 in the placebo group) were lost at follow-up since the trial was interrupted by the outbreak of the COVID-19 pandemic that caused problems with nutritional coaching in person.

### 3.1. Results by Randomization

**Figure 1:**
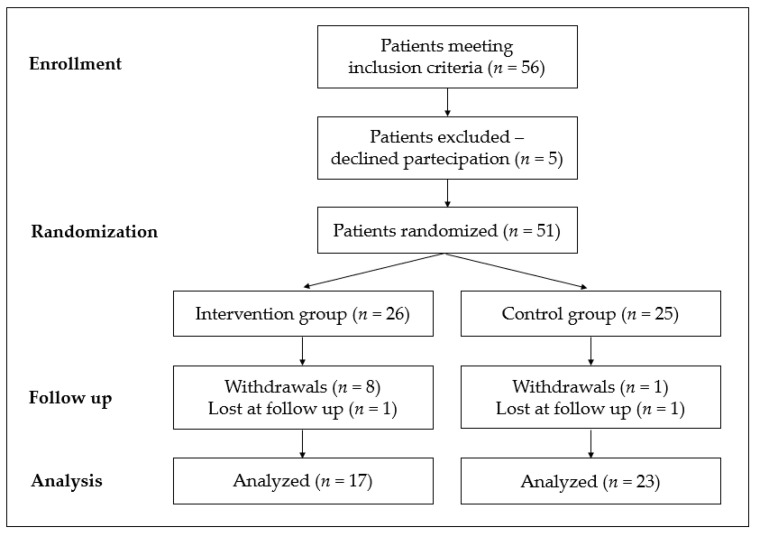
Study enrollment and randomization.

Forty women completed the study protocol, of whom 17 were assigned to the intervention group and 23 to the placebo group by randomization (Figure 1). There were no significant differences as regards age, anthropometric, metabolic, and inflammatory parameters between the two groups at enrollment (Table S1). No significant differences were observed for the same indices after 12 weeks of treatment. In addition, there were no differences between the two groups in maternal and fetal outcomes, including infant weight and birth weight percentile (Table 1).

However, 4 out of 17 women in the intervention group (23.5%) and 2 out of 23 in the control group (8.7%) required pharmacological therapy to regulate blood glucose levels.

In the intervention group, 12 out of 17 enrolled subjects (70.6%) showed a reduction in the RBC AA/EPA ratio at the end of the study, while in the placebo group, 12 out of 23 enrolled patients (52.2%) showed a reduction in the AA/EPA ratio (Chi-square 1.38, *p* = 0.240) (Figure S1, panel A); as described by other authors, these data indicate poor compliance with the integration protocol [28]. PAF values were not significantly different neither at T0 nor at T12.

### 3.2. Results by Severity of GD

Data at T0 of all the subjects enrolled were analyzed as a predictor of severity.

Of the six patients who required pharmacological intervention, 5 out of 6 (83.3%) presented with alterations in basal glycemia, while 2 (33.3%) presented with alterations in post-prandial glycemia. It took up to 8 weeks to reach a glucose control.

Patients with GD who required insulin therapy had a significantly higher BMI and pre-pregnancy weight, subscapular fold, bicipital fold, and triceps fold, as well as arm circumference and wrist circumference at T0 than patients who did not (Table 2).

Subjects who needed pharmacological intervention showed higher plasma triglycerides, CRP levels, and insulin levels at the time of diagnosis than subjects who did not require drug therapy (Table 3).

**Figure 2:**
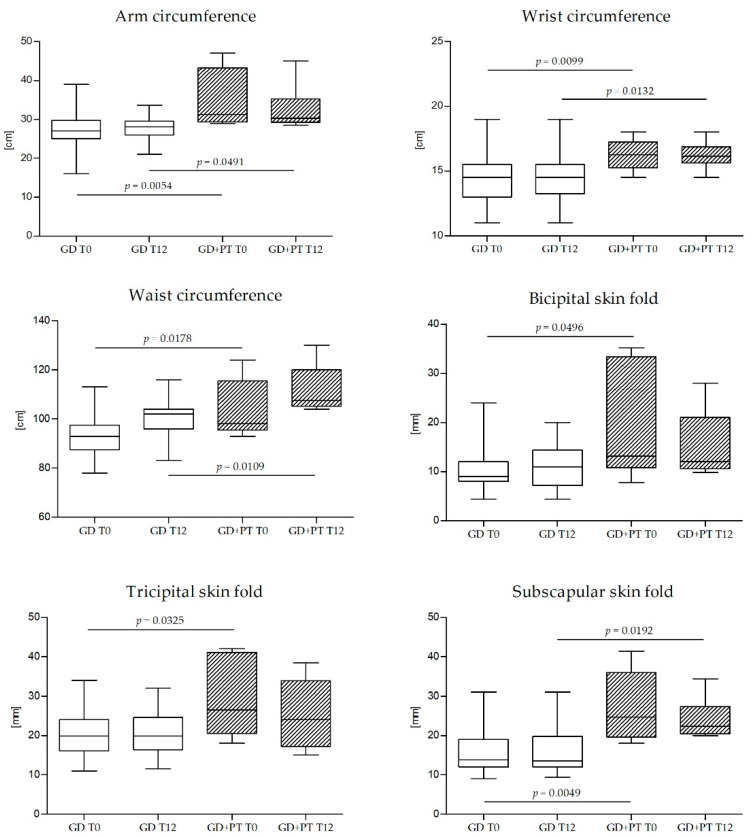
Longitudinal trend of anthropometric parameters of subjects with GD who required therapy to normalize blood glucose levels (GD+PT) at enrollment (T0; *n* = 6) and at and the end of the study after 12 weeks (T12; *n* = 6) with subjects who did not require pharmacological intervention (GD) at T0 (*n* = 45) and at T12 (*n* = 34). GD—subjects with gestational diabetes mellitus who did not require pharmacological therapy. GD+PT—subjects with gestational diabetes mellitus who required pharmacological therapy. T0—at enrolment. T12—after 12 weeks.

**Figure 3:**
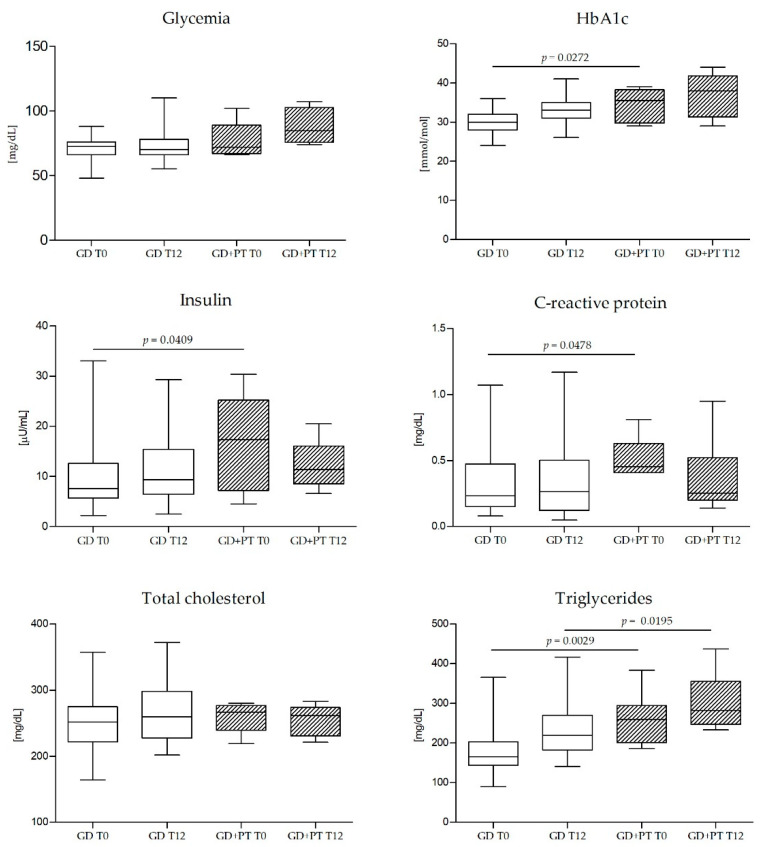
Longitudinal trend of plasmatic metabolic and inflammatory parameters of subjects with GD who required therapy to normalize blood glucose levels (GD+PT) at enrollment (T0; *n* = 6) and at the end of the study after 12 weeks (T12; *n* = 6) with subjects who did not require pharmacological intervention (GD) at T0 (*n* = 45) and at T12 (*n* = 34). GD—subjects with gestational diabetes mellitus who did not require pharmacological therapy. GD+PT—subjects with gestational diabetes mellitus who required pharmacological therapy. T0—at enrollment. T12—after 12 weeks.

Subscapular fold and arm circumference in patients who needed insulin remained significantly greater even after 12 weeks of treatment (Figure 2). In this group, triglyceride levels were even higher twelve weeks after diagnosis compared with subjects on diet therapy alone (Figure 3).

Infants born to women with GD on drug treatment had a higher birth weight and birth weight percentile than infants born to women with GD who did not require medications (3855 g (IQR 3425–4090) vs. 3165 g (3013–3404) (*p* < 0.01) and 92nd percentile (IQR 62–96) vs. 35th percentile (IQR 18–61) (*p* < 0.001), respectively).

To assess the influence of the PUFA levels on the predisposition to the development of complicated GD, we compared subjects within the lower quartile of AA/EPA ratio in RBC at T0 (*n* = 13; AA/EPA < 28.7; Low AA/EPA) with subjects within the higher AA/EPA ratio quartile (*n* = 13; AA/EPA > 55.2; High AA/EPA) (Figure S1, panel B).

In the quartile with the lowest AA/EPA ratio, there was only one case of GD in which it was necessary to use drug therapy. In contrast, in the quartile with a higher AA/EPA ratio, there were 3 out of 6.

**Figure 4:**
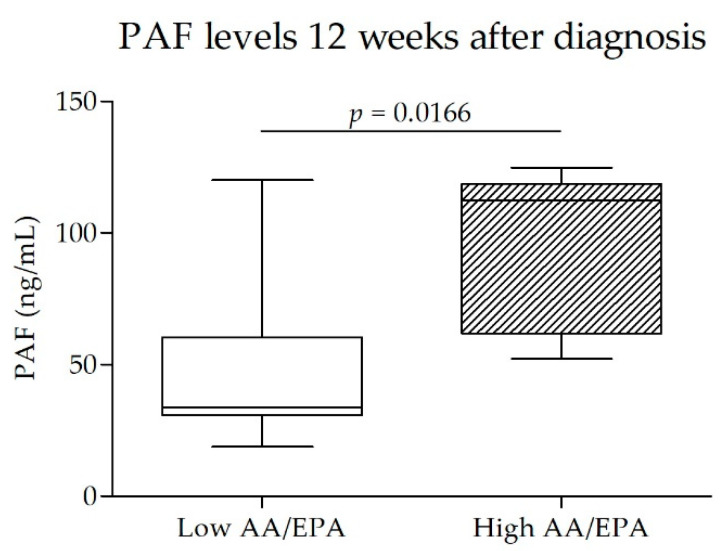
Comparison of PAF levels at the end of the study (after 12 weeks from randomization; T12) between subjects within the lower quartile of AA/EPA ratio in red blood cells at enrollment (*n* = 13; AA/EPA < 28.7; Low AA/EPA) and subjects within the higher AA/EPA ratio quartile (*n* = 13; AA/EPA> 55.2; High AA/EPA). AA/EPA—arachidonic acid/eicosapentaenoic acid ratio.

Subjects with a lower AA/EPA ratio 12 weeks after the diagnosis of GD had lower PAF levels than subjects with a higher AA/EPA ratio (*p* < 0.05) (Figure 4).

**Figure 5:**
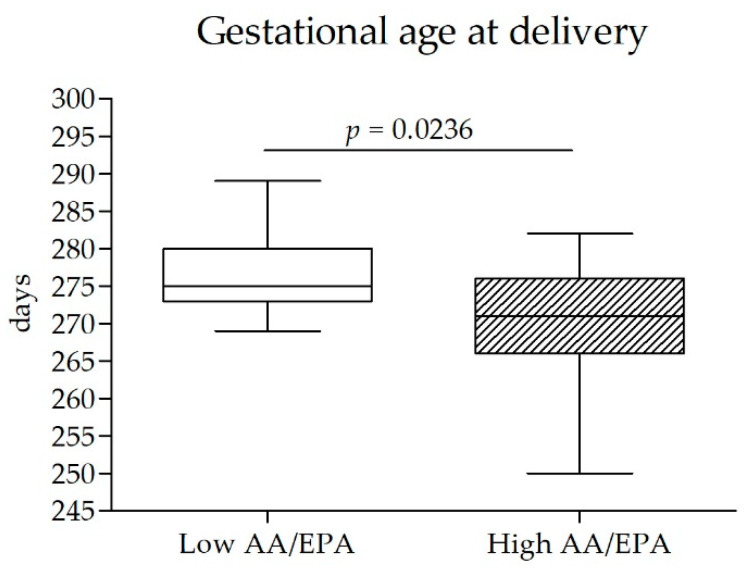
Comparison of the gestational age at delivery between subjects within the lower quartile of AA/EPA ratio in red blood cells at enrollment (*n* = 13; AA/EPA < 28.7; Low AA/EPA) and subjects within the higher AA/EPA ratio quartile (*n* = 13; AA/EPA > 55.2; High AA/EPA). AA/EPA—arachidonic acid/eicosapentaenoic acid ratio.

Subjects with a higher AA/EPA ratio gave birth four days earlier than subjects with a lower AA/EPA ratio (*p* < 0.05) (Figure 5).

## 4. Discussion

Our randomized double-blind trial analyzed key maternal metabolic and anthropometric data, gestational age at delivery, and neonatal birth weight between pregnant patients affected by GD receiving nutraceutical supplementation with omega-3 fatty acids, anthocyanins, and alpha-cyclodextrins, or placebo. Both arms of this randomized study underwent a strict dietary follow-up by a nutrition expert educator; this approach supported in a one-to-one manner a diet which increased the intake of food rich in omega-3 fatty acids, reducing the AA/EPA ratio and paring the effect of nutraceutical supplements. No statistically significant differences were observed between the groups in blood and urine measurements of metabolic, inflammatory, and antioxidant parameters.

Two factors likely influenced these results. Firstly, differences could have been covered by the expected effect of the diet, emphasizing its positive effects when multiple assessments of adherence and personalized counseling are provided to patients [29]. Opposite to this, we reported poor adherence to the supplementation protocol, as shown by the analysis of the AA/EPA ratio changes in RBC from enrollment to the end of the study.

### 4.1. Maternal Anthropometric Parameters

Independently from allocation in the treatment and control arms, our study shows that GD patients requiring pharmacological therapy were characterized by different maternal anthropometric and metabolic baseline features. These subjects presented a greater BMI and pre-pregnancy weight, in agreement with current evidence [30,31,32]. Moreover, subscapular fold, bicipital fold, triceps fold, arm circumference, and wrist circumference were greater at the time of diagnosis, and this difference persisted even 12 weeks after diagnosis for subscapular fold and arm circumference.

Lately, since gestational diabetes displays a growing health impact and represents a public health challenge, greater interest has been shown in developing risk stratification and risk-based models of care to predict the need for pharmacological therapy and choose an adequate one [33,34,35].

Our study suggests that including skinfold thickness assessment is an easy, cost-effective tool to use in routine clinical examinations to quantify body fat percentage and describe its distribution. It only requires training to be highly reliable and reproducible. This evaluation could be exploited in addition to the assessment of body composition by bioimpedentional tools, in place of dual-energy X-ray absorptiometry [36], which is contraindicated during pregnancy and more expensive [37]. However, this approach is limited by high observer variability, which can be reduced only by following rigorous training and expertise [38,39].

### 4.2. Maternal Triglycerides

Triglycerides, glycated hemoglobin, insulin, and CRP confirmed their role as valuable predictors of the need for pharmacological hypoglycemic treatment [40].

An interesting finding of our study is that triglyceride levels were still higher at term in subjects requiring pharmacological therapy for GD, compared to those on diet therapy alone, independently from the arm of randomization and glycemic control. Hypertriglyceridemia has been related to impaired insulin action and β-cell function in pregnancy. This molecular mechanism has been associated with a higher risk of developing GD, even in the case of moderately elevated levels [41,42,43]. However, to our knowledge, there is insufficient evidence on the effects of standard treatment for diabetes on hypertriglyceridemia [44]. An exciting path for future research could be the inclusion of lipid profiles in subjects who present accelerated fetal growth despite good glycemic control achieved through therapy. The evidence of a slight increase in these values considered altogether could help to identify candidates for therapy or to accelerate decision-making in uncertain contexts.

### 4.3. Maternal AA/EPA Ratio and PAF

The AA/EPA ratio appears to predict the metabolic dysfunction of subjects affected by GD. A high AA/EPA ratio at the time of diagnosis of GD is associated with an increase in PAF levels and a greater likelihood of drug therapy in addition to a well-controlled diet. PAF levels have proved to be inflammatory biomarkers in women with GD [22].

Interestingly, the balance between omega-3 and omega-6 fatty acids also influences gestational age at delivery. A more inflammatory condition appears to anticipate delivery. If confirmed, this data could lead to the introduction of the evaluation of the AA/EPA ratio for a better estimate of the term of gestation.

Triglycerides and AA/EPA values were associated with the need for drug therapy in addition to the diet. Moreover, in spite of good glycemic control, newborns from these subjects were significantly heavier than newborns of mothers who achieved a better lipogram profile.

### 4.4. Limitations

The limited size of the two groups represents the most significant limitation of our work; the outbreak of the COVID-19 pandemic prevented us from reaching the planned sample size for each arm of the trial. Further studies with a larger cohort are needed to confirm our results.

Our study population was represented mainly by Caucasian women due to the usual referrals to the hospital involved. Moreover, pre-conception BMI was not considered an exclusion criterion.

In addition, in our study, the glycemic control assessment included only changes in hematic glycated hemoglobin and the proportion of patients presenting median blood glucose values 10 mg/dL below the target at the end of the study. Current evidence suggests that slight differences in response to nutritional and environmental factors are known to determine epigenetic modifications that could influence fetal metabolic programming [15]. Moreover, recent studies reveal that fetuses of GD women present alterations compared to controls even in the case of minimal glycemic changes, which are undetectable with standard clinical tests [45]. This is in line with the significant role of triglycerides and AA/EPA ratio observed in our study as regards both the need for insulin and newborn weight.

## 5. Conclusions

The effects of supplementation with omega-3 and antioxidants on metabolic and inflammatory parameters of women with GD were not significant compared with strict and one-to-one careful nutritional consultation that paired the effects of supplementation.

The role of the nutritional counselor and the role of doctors in charge of supporting additional therapies appeared to be of major importance as regards compliance with nutritional recommendations and nutraceutical support.

Remarkable differences were found when comparing subjects with GD who needed supportive pharmacological therapy with those who did not. In the former group, the anthropometric and metabolic parameters, particularly the skin folds and the level of triglycerides, and the AA/EPA ratio, were significantly altered in early gestation. Our preliminary data suggest that these parameters could be used for the early identification of GD subjects with higher inflammatory levels and likely candidates for pharmacological treatment, although this should be confirmed by further studies on larger cohorts. Adipose tissue is per se a proinflammatory organ and its key role in the severity of metabolic syndrome was observed in this cohort of pregnant women with gestational diabetes. Indeed, an adequate assumption of omega-3 in women with GD, either by a controlled diet or by nutraceutical supplementation, reduces the need for pharmacological therapy.

## Figures and Tables

**Table 1:** Anthropometric, metabolic, and inflammatory parameters at T12 of the intervention group and the placebo group.

	IG T12 (*N* = 17)	PG T12 (*N* = 23)	*p*
Arm circumference (cm)	28.6 (26.8–29.3)	28.5 (28.0–30.0)	0.2202
Wrist circumference (cm)	15 (14.5–15.5)	15 (14.5–16.0)	0.3312
Waist circumference (cm)	94.5 (91–99)	97 (93.0–102)	0.1590
Bicipital skin fold (mm)	8.5 (7.3–10.3)	10.4 (7.7.15.8)	0.3162
Triceps skin fold (mm)	20.0 (19.0–23.4)	23.8 (18.4–27.3)	0.1195
Subscapular skin fold (mm)	15.8 (14.4–19.0)	16.4 (14.4–24)	0.2795
Glycemia (mg/dL)	72 (68–82)	71 (66–78)	0.6073
HbA1c (mmol/mol)	33 (32–36)	33 (31–34)	0.2034
Insulin (µU/mL)	9.7 (7.4–14.9)	9.3 (5.8–15.1)	0.5333
Total cholesterol (mg/dL)	269 (228–313)	250 (221–278)	0.4722
LDL cholesterol (mg/dL)	146 (114–188)	126 (106–154)	0.1166
HDL cholesterol (mg/dL)	69 (57–80)	73 (65–87)	0.5457
Triglycerides (mg/dL)	229 (181–271)	227 (195–283)	0.5490
RBC AA/EPA ratio	17.2 (11.1–30.1)	24.2 (14.9–40.2)	0.5716
Cortisol (µg/L)	29.8 (22.8–38.5)	26.3 (23.4–32.9)	0.5849
C-reactive protein (mg/dL)	0.27 (0.19–0.47)	0.23 (0.17–0.52)	0.8691
PAF (ng/dL)	33.9 (29.1–51.2)	37.7 (31.3–56.8)	0.4160

IG—intervention group. PG—placebo group. LDL—low density lipoprotein; HDL—high-density lipoprotein. RBC AA/EPA ratio—arachidonic acid/eicosapentaenoic acid ratio in cell membranes of erythrocytes. PAF—platelet-activating factor. T12—after 12 weeks from the diagnosis of GD.

**Table 2:** Age and anthropometric parameters at T0 of subjects with GD who required therapy to normalize blood glucose levels (GD+PT) and subjects who did not require pharmacological intervention (GD).

	GD (*N* = 45)	GD+PT (*N* = 6)	*p*
Age (years)	34 (32–37)	36 (32–38)	0.5985
Height (cm)	163 (160–170)	165 (162–167)	0.6358
Pre-pregnancy weight (kg)	60.0 (52.5–68.0)	69.5 (65.3–103)	**0.0149**
Pre-pregnancy BMI (kg/m^2^)	22.0 (20.1–24.0)	25.8 (24.5–37.2)	**0.0043**
Arm circumference (cm)	27.0 (25.0–29.8)	31.3 (29.4–43.3)	**0.0054**
Wrist circumference (cm)	14.5 (13–15.5)	16.25 (15.25–17.25)	**0.0099**
Waist circumference (cm)	92.0 (87.0–95.6)	98.0 (95.5–115.5)	**0.0178**
Bicipital skin fold (mm)	9.0 (8.0–12.0)	13.2 (10.8–33.4)	**0.0496**
Triceps skin fold (mm)	19.9 (16.1–24.0)	26.5 (20.6–40.9)	**0.0325**
Subscapular skin fold (mm)	13.8 (12.0–19.0)	24.7 (19.7–36.0)	**0.0049**

GD—subjects with gestational diabetes mellitus who did not require pharmacological therapy. GD+PT—subjects with gestational diabetes mellitus who required pharmacological therapy. Bold font highlights statistical significance (*p* < 0.05).

**Table 3:** Metabolic and inflammatory parameters at T0 of subjects with GD who required therapy to normalize blood glucose levels (GD+PT) and subjects who did not require pharmacological intervention (GD).

	GD (*N* = 45)	GD+PT (*N* = 6)	*p*
Glycemia (mg/dL)	73 (66–76)	74 (67–89)	0.2683
HbA1c (mmol/mol)	30 (28–32)	36 (30–38)	**0.0272**
Insulin (µU/mL)	7.5 (5.7–12.6)	17.3 (7.2–25.2)	**0.0409**
Total cholesterol (mg/dL)	252 (222–275)	267 (239–277)	0.2819
LDL cholesterol (mg/dL)	140 (109–162)	135 (115–171)	0.5158
HDL cholesterol (mg/dL)	80 (66–88)	60 (50–88)	0.0865
Triglycerides (mg/dL)	165 (144–203)	260 (201–295)	**0.0029**
C-reactive protein (mg/dL)	0.24 (0.15–0.49)	0.5 (0.41–0.63)	**0.0478**
Cortisol (µg/L)	28.6 (22.8–31.9)	24.8 (20.5–29.0)	0.1230

GD—subjects with gestational diabetes mellitus who did not require pharmacological therapy. GD+PT—subjects with gestational diabetes mellitus who required pharmacological therapy. Bold font highlights statistical significance (*p* < 0.05).

## Data Availability

Not applicable.

## Supplementary Materials

The following supporting information can be downloaded at: https://www.mdpi.com/article/10.3390/nu14122502/s1, Table S1: Age, anthropometric, metabolic, and inflammatory parameters, birth weight and birth weight percentile of the intervention group and the placebo group. Figure S1: The plot of individual AA/EPA ratio in the intervention group (IG) (n = 17) and in the placebo group (PG) (n = 23) at T0 and T12 (panel A); quartiles distribution of individual AA/EPA ratio at T0 (panel B).
