# Supplementary material for: Maternal AA/EPA Ratio and Triglycerides as Potential Biomarkers of Patients at Major Risk for Pharmacological Therapy in Gestational Diabetes
Source: Nutrients. 2022 Jun 16;14(12):2502. doi: 10.3390/nu14122502 (PMC9231064; doi:10.3390/nu14122502)
Supplement: Supplementary file 1 [file nutrients-14-02502-s001.zip › nutrients-1746419-supplementary.pdf]

Table S1. Age, anthropometric, metabolic, and inflammatory parameters, birth weight and birth weight percentile of the intervention group and the placebo group.

	IG (N=17)	PG (N=23)	p
Age (years)	34 (32-37)	34 (33-38)	0.9500
Height (cm)	161 (159-165)	163 (160-167)	0.3172
Pre-pregnancy weight (kg)	58.8 (51.5-66.0)	62.0 (56.5-68.5)	0.1256
Pre-pregnancy BMI (kg/m^2)	22.4 (20.2 -23.7)	23.3 (21.0-24.0)	0.5556
Birth weight (g)	3250 (3073-3428)	3215 (2933-3050)	0.8139
Birth weight percentile	34 (20-64)	33 (12-67)	0.9258
	**IG T0 (N=17)**	**PG T0 (N=23)**	**p**
Arm circumference (cm)	28.5 (26.4-29.6)	29.0 (28.1-30.3)	0.4446
Wrist circumference (cm)	15.0 (12.8-15.5)	14.5 (13.5-16)	0.5678
Waist circumference (cm)	92.5 (87.4-97.3)	93 (91.8-99.0)	0.6780
Bicipital skin fold (mm)	9.9 (7.7-12.3)	10.0 (8.7-14.8)	0.4026
Triceps skin fold (mm)	19.6 (16.8-21.7)	22 (19.8-24.4)	0.1544
Subscapular skin fold (mm)	16.5 (13-19.3)	18.4 (14.0-25.8)	0.1782
Glycemia (mg/dL)	73 (67-77)	72 (66-75)	0.5091
HbA1c (mmol/mol)	31 (30-32)	30 (28-31)	0.2424
Insulin (μU/mL)	7.1 (5.2-14.2)	9.5 (6.2-14.3)	0.3868
Total cholesterol (mg/dL)	265 (226-279)	230 (218-275)	0.2935
LDL cholesterol (mg/dL)	122 (103-152)	146 (144-188)	0.1034
HDL cholesterol (mg/dL)	73 (64-85)	81 (65-88)	0.6220
Triglycerides (mg/dL)	182 (128-213)	175 (148-211)	0.9508
RBC AA/EPA ratio	16.9 (10.2-25.0)	22.3 (13.8-33.1)	0.1574
Cortisol (μg/L)	29.2 (25.2-32-8)	27.4 (21.1-31.0)	0.2966
C-reactive protein (mg/dL)	0.33 (0.16-0.43)	0.21 (0.13-0.54)	0.3879
PAF (ng/dL)	23.4 (17.3-36.9)	30.4 (17.9-48.3)	0.6424

IG = Intervention Group. PG = Placebo Group. RBC AA/EPA ratio = arachidonic acid/eicosapentaenoico acid ratio in cell membranes of erythrocytes. PAF = Platelet-activating factor. T12 = after 12 weeks from the diagnosis of GD. ns = p > 0.05.

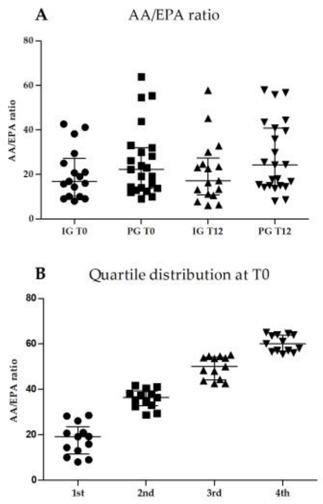

Figure S1. Individual AA/EPA ratio in the intervention group (IG) (n=17) and in the placebo group (PG) (n=23) at T0 and T12 (panel A); quartiles distribution of individual AA/EPA ratio at T0 (panel B).
